# Interleukin-21 Is a Critical Regulator of CD4 and CD8 T Cell Survival during Priming under Interleukin-2 Deprivation Conditions

**DOI:** 10.1371/journal.pone.0085882

**Published:** 2014-01-09

**Authors:** Mithun Khattar, Yoshihiro Miyahara, Paul M. Schroder, Aini Xie, Wenhao Chen, Stanislaw M. Stepkowski

**Affiliations:** 1 Department of Medical Microbiology and Immunology, University of Toledo College of Medicine, Toledo, Ohio, United States of America; 2 Transplant Immunology Center, Houston Methodist Research Institute, Houston, Texas, United States of America; University of Ottawa, Canada

## Abstract

Optimal T cell activation and expansion require binding of the common gamma-chain (γc) cytokine Interleukin-2 (IL-2) to its cognate receptor that in turn engages a γc/Janus tyrosine kinase (Jak)3 signaling pathway. Because of its restricted expression by antigen-activated T cells and its obligatory role in promoting their survival and proliferation, IL-2 has been considered as a selective therapeutic target for preventing T cell mediated diseases. However, in order to further explore IL-2 targeted therapy, it is critical to precisely understand its role during early events of T cell activation. In this study, we delineate the role of IL-2 and other γc cytokines in promoting the survival of CD4 and CD8 T cells during early phases of priming. Under IL-2 inhibitory conditions (by neutralizing anti-IL-2 mAbs), the survival of activated CD8^+^ T cells was reduced, whereas CD4^+^ T cells remained much more resistant. These results correlated with reduced Bcl-2 expression, and mitochondrial membrane potential in CD8^+^ T cells in comparison to CD4^+^ T cells. However, using transwell co-culture assays we have found that CD4^+^ T cells could rescue the survival of CD8^+^ T cells even under IL-2 deprived conditions via secretion of soluble factors. A cytokine screen performed on CD8^+^ T cells cultured alone revealed that IL-21, another γc cytokine, was capable of rescuing their survival under IL-2 deprivation. Indeed, blocking the IL-21 signaling pathway along with IL-2 neutralization resulted in significantly reduced survival of both CD4^+^ and CD8^+^ T cells. Taken together, we have shown that under IL-2 deprivation conditions, IL-21 may act as the major survival factor promoting T cell immune responses. Thus, investigation of IL-2 targeted therapies may need to be revisited to consider blockade of the IL-21 signaling pathways as an adjunct to provide more effective control of T cell immune responses.

## Introduction

T cells play a central role in cell mediated immune responses to foreign antigens *via* recognition through their T cell receptors (TCR). In addition to TCR signals, optimal T cell activation and expansion require co-stimulatory and cytokine signals. The cytokine signals leading to T cell activation and proliferation involve binding of common γ-chain (γc) cytokines (interleukin (IL)-2, IL-4, IL-7, IL-9, IL-15 and IL-21) to their cognate receptors which in-turn activates Janus tyrosine kinases (Jak) 1 or Jak3 in the downstream milieu inducing transcription of multiple genes through signal transducers and activators of transcription (Stat)3, Stat6 and Stat5a/b pathways [1]. Among these cytokines, IL-2 is the major growth factor optimizing T cell responses as signaling through its high affinity IL-2 receptor (consisting of the α, β and common γ chains) and the Jak3-Stat5 axis is essential for the survival, proliferation and differentiation of antigen-activated T cells [2]–[5]. Naïve and memory T cells lack IL-2Rα (CD25) expression, but its expression is induced soon after antigen activation. Once the high affinity IL-2R is induced, IL-2 signaling upregulates Jak3-Stat5 mediated *il2ra* transcription, and hence maintains CD25 expression and IL-2 signaling as long as a source of IL-2 is present [6]. IL-2 is exclusively produced by effector CD4 and CD8 T cells upon antigen induced activation. During an ongoing immune response, this IL-2 is utilized in an autocrine and paracrine fashion by activated cells in close proximity which leads to activation of the MAPK and PI-3K pathways, facilitating the expansion of effector CD4 and CD8 T cells [7]. Once the optimal threshold of cellular proliferation for an effective immune response is achieved, IL-2 transcription is repressed in activated T cells by T-bet and Blimp-1 to limit the unrestrained expansion of antigen-reactive T cells [8]–[10]. In addition to its proliferative function in effector T cells, IL-2 also regulates several aspects of T helper (Th) and memory cell differentiation. IL-2 is essential for induction of both effector Th1 and Th2 cells in a STAT5 dependent manner [11], [12]. Further, IL-2 inhibits T helper17 (Th17) [13], [14] and T follicular helper (T_FH_) [15], [16] cell differentiation, but more recent reports show that IL-2 can expand the Th17 cells once generated, thus exerting complex actions on Th17 differentiation [17]. Besides its actions on Th cell populations, IL-2 also drives the development of naive CD8 T cells into memory cytolytic T lymphocytes (CTL) upon antigen stimulation [18], [19]. Because of its critical role in driving effector and memory T cell survival, proliferation and differentiation as well as its exclusive transient expression in antigen-activated T cells, IL-2 has been considered as a potential therapeutic target for modulating the immune response. For instance, several Jak3 inhibitors to block IL-2 signaling have been designed for promoting immunosuppression and transplantation tolerance [20], [21]. Similarly, IL-2R blockade using monoclonal antibodies (mAbs) Daclizumab and Basiliximab have also been explored as induction immune therapies. However, the widespread clinical use of these inhibitors and mAbs is discouraged due to limited efficacy and excessive side effects in preclinical and clinical transplantation models [22]–[25]. Thus, we need to better understand the IL-2 dependent and independent functions of different T cell subsets and then apply this knowledge for designing better therapies to achieve successful immunosuppression and even induction of tolerance.

IL-2 signaling via Jak3-Stat5a/b also controls the generation, maintenance and function of FoxP3^+^ regulatory T cells (Tregs) as Tregs constitutively express the high affinity IL-2R [4], [26]–[28]. Deficiency of IL-2, IL-2Rα, or IL-2Rβ in mice leads to development of lethal autoimmune diseases, which has been attributed to defects in IL-2 signaling within the Treg cells [29]–[31]. These findings were surprising as deficiency in IL-2 signaling in effector T cells did not result in loss of immunity, but rather lead to rapid and lethal autoimmunity. Further studies using IL-2 and IL-2R deficient mice reconstituted with functional Tregs have revealed that expansion of activated T cells can occur without IL-2 signaling [4], [32]. What accounts for this IL-2 independent function of T cells is not yet established and thus, there is a clear need to investigate the critical growth factors that promote the survival and function of activated T cells under IL-2 deprived conditions.

IL-21 is the most recently discovered common-γ chain cytokine that has been shown to play critical roles in both innate and adaptive immunity [33]. Although IL-21 is predominantly produced by effector CD4^+^ T cells, its receptor (IL-21R) is more broadly expressed on T, B, NK and dendritic cells (DCs) and has direct pleiotropic effects on their proliferation, differentiation and effector functions [33], [34]. Particularly, during chronic LCMV infections when IL-2 production by T cells is decreasing, prolonged IL-21 production by antigen-specific CD4^+^ Th cells is needed to sustain long-term effector functions of CD8^+^ T cells [35]–[37]. Similarly, in non-obese diabetic (NOD) mice which have genetic polymorphisms in the *il2* gene locus and lower IL-2 production, IL-21 plays a critical role in sustaining pathogenic auto-immune responses that lead to development of type-1 diabetes (T1D) in these mice [38]–[40]. Thus, IL-21 seems to be a key regulator of chronic T cell responses when IL-2 production by T cells is shut down either naturally or by genetic changes. However, whether IL-21 plays a role in promoting the survival of T cells during the early events of priming under IL-2 deprivation is currently unknown. Addressing these questions will improve our understanding of the cytokine signaling in T cell mediated immune responses and will assist in designing better immune therapies.

In the present study, we have investigated the role of IL-2 dependent and independent signals on promoting the survival of activated CD4^+^ and CD8^+^ T cells during priming. We have found that blockade of IL-2 signaling affected predominantly the survival of CD8^+^ T cells in individual cultures, whereas when co-cultured together, CD4^+^ T cells rescued the survival of CD8^+^ T cells under IL-2 deprivation through soluble factors. IL-21 was found to be one of the critical factors mediating this help from CD4^+^ to CD8^+^ T cells. These results suggest alternate patterns of cytokine signaling to promote T cell survival and function in the absence of IL-2.

## Materials and Methods

### Animals

C57BL/6 (B6) and C57BL/6-Tg(BCL2)25Wehi/J (Bcl-2 Tg) mice were purchased from Jackson Laboratories (Bar Harbor, ME). B6.129S-*Il21^tm1Lex^*/Mmcd (IL-21 KO) mice were obtained from Mutant Mice Regional Resource Center (MMRRC), Davis, CA. Mice were 8–12 weeks old either males or females which were age and sex matched when euthanized for each experiment. All mice were used according to institutional guidelines.

### Ethics Statement

Animal work was performed in accordance with the Guide for the Care and Use of Laboratory Animals of the National Research Council. Animals were maintained at the University of Toledo Health Science Campus specific pathogen-free animal facility according to institutional guidelines. These studies were performed as described in a protocol approved by the University of Toledo Health Science Campus Institutional Animal Care and Use Committee (IACUC) (Protocol number 105921). Mice were euthanized by CO_2_ asphyxiation followed by cervical dislocation.

### Cytokines, Reagents, and Antibodies

Recombinant mouse cytokines were obtained from R&D Systems. Propidium Iodide (PI) was obtained from Sigma-Aldrich. FITC-conjugated anti-mouse CD4 (clone GK1.5), APC-conjugated anti-CD8 (Ly-2) and PE-conjugated anti-CD25 (PC61) mAbs were purchased from eBioscience. Purified anti-CD3ε (145-2C11), anti-CD28 (37.51), anti-mouse-IL-2(S4-B6) and anti-mouse Bcl2 staining kit were purchased from BD Biosciences. Mitotracker Red staining kit to measure mitochondrial membrane potential and anti-CD3/CD28 coated beads were purchased from Molecular Probes (Invitrogen).

### Cell Survival Assay and Staining

Single cell suspensions were obtained from spleens of 8–10 week old wild-type B6 or IL-21 KO mice as indicated in text and figures, and were stained with anti-CD4-FITC, anti-CD8-APC and anti-CD25-PE. CD4^+^CD25^−^ and CD8^+^CD25^−^ cells were sorted on a FACSAriaIII cell sorter (BD Biosciences) and were cultured in 96-well plates at 5×10^5^ cells per well in RPMI 1640 supplemented with 10% FCS on plate-bound anti-CD3 (4 µg/mL) and soluble anti-CD28 (2 µg/mL) mAbs without or with neutralizing IL-2 antibodies at two different concentrations i.e., 1 µg/mL and 10 µg/mL, as indicated in figures. For co-culture experiments in 96-well plates, 2.5×10^5^ cells from each population were seeded per well. In some experiments, cells were cultured without stimulation (i.e. in absence of anti-CD3 and anti-CD28 Abs) as negative controls. In the cytokine screening experiment, CD8^+^ T cell cultures with 10 µg/ml anti-IL-2 were further supplemented with either 10 IU/mL (IL-2) or 10 ng/mL (all other cytokines); each of 61 different cytokines. The concentration of 10 ng/mL for all cytokines was selected based on the known effectiveness of certain T cell stimulating cytokines to promote T cell survival, proliferation and differentiation in vitro [41]. Cultured cells were harvested at different time points in cultures as indicated in the figures and subsequently stained with 1 µg/mL PI for measuring cell survival. Bcl2-PE and Mitotracker Red dye intracellular staining were performed as described in the respective intracellular staining kits. The cells were subsequently analyzed on a FACS Calibur.

### Trans-well Experiments

CD4^+^CD25**^−^** T cells (1×10^5^) were isolated from B6 or IL-21 KO mice, and were cultured in the upper chamber of the 24-well transwell plates (Corning, NY, USA). CD8^+^CD25**^−^** (1×10^5^) T cells from B6 or IL-21 KO mice were placed in the bottom chamber of some transwells. Both populations of cells in upper and lower chambers were activated with Dynabeads Mouse T-activator CD3/CD28 (Invitrogen; bead-to-cell ratio = 1∶1). As one of the controls, both CD4^+^CD25**^−^** and CD8^+^CD25**^−^** T cells (1×10^5^) were added into the upper chamber of some transwells. Where indicated, IL-2 neutralizing mAb was added to the cultures. Cultured cells were harvested at 48 hrs and subsequently stained with 1 µg/mL PI for measuring cell survival.

### ELISA

Supernatants were collected from the cultures at 24 and 48 hrs as indicated. The levels of IL-2 in culture supernatants were then assessed by ELISA using “Mouse IL-2 Duoset” ELISA development kit from R & D Systems (Minneapolis, MN) following the assay procedure provided with manufacturer’s instructions.

### Statistical Analysis

All statistical analyses were performed using an unpaired Student’s t-test to determine statistical significance at the levels indicated in the text.

## Results

### Neutralizing IL-2 mAbs More Effectively Block the Survival of Activated CD8^+^ than CD4^+^ T Cells

**Figure 1:**
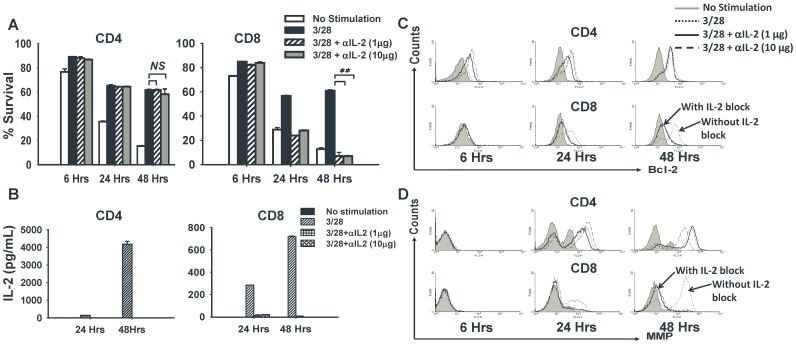
Neutralizing IL-2 reduces the survival of CD8^+^ but not CD4^+^ T cells. CD4^+^CD25**^−^** and CD8^+^CD25**^−^** T cells were purified from wild-type B6 mice and stimulated with anti-CD3/CD28 mAbs in the presence or absence of neutralizing IL-2 mAb at two different concentrations as indicated. Some cells were left unstimulated (no anti-CD3/CD28) and were used as negative controls. A total of 5×10^4^ cells per well were cultured in flat-bottom 96-well plates and harvested for analysis at the indicated time points. (**A**) Harvested cells were stained with Propidium Iodide (PI) to measure cell survival by flow cytometry. Bar graphs show the percentage of PI negative cells in each group (n = 3). (**B**) Supernatants were harvested from the cultures at the indicated time points which were then analyzed for levels of IL-2 using ELISA. Bar graphs show the concentration of IL-2 in the culture supernatants. (**C**) Histograms show the expression of the anti-apoptotic protein Bcl-2 measured by flow cytometry in the cultured cells at the indicated time points. (**D**) Histograms represent the mitochondrial membrane potential measured by flow cytometry of harvested cells (with higher fluorescence depicting a hyper-polarized state of mitochondria) at the indicated time points. The figure shows results from one representative experiment which was repeated three times with similar results. Bars indicate average value ± standard deviation (SD) obtained from three culture wells (technical triplicates) in each experimental group (*denotes *p*<0.05 and **denotes *p*<0.005).

T cell activation requires signals from T cell receptor (TCR), co-stimulatory molecules B7/CD28, as well as from γc-cytokines through their receptors that activate the Jak3/Stats pathways [1]. In this study, we examined the impact of early deficiency of the major proliferative cytokine IL-2 on the survival of activated CD4^+^ versus CD8^+^ cells during the first 48 hrs of cultures. Naïve CD4^+^ and CD8^+^ T cells were stimulated *in vitro* with CD3 and CD28 mAbs and cultured in the presence (or absence) of two different concentrations of a neutralizing IL-2 mAb (clone S4-B6) [27], [41], [42]. As shown by the PI staining, survival of CD4^+^ T cells was not affected by neutralization of IL-2 during activation, whereas CD8^+^ T cells showed significantly reduced survival (Fig. 1A). Both lower and higher concentrations of anti-IL-2 mAb reduced survivals of activated CD8^+^ T cells by 50% at 24 hrs and by 90% at 48 hrs (Fig. 1A). We confirmed the neutralization of IL-2 in these cultures by measuring IL-2 levels in the supernatants. There was no detectable IL-2 in the CD4^+^ and CD8^+^ T cell cultures supplemented with either 1 µg/mL or 10 µg/mL of anti-IL-2, thus validating complete inhibition of IL-2 signaling (Fig. 1B). These results correlated with the expression of anti-apoptotic protein Bcl-2. The cultured CD8^+^ T cells displayed reduced Bcl-2 expression, whereas CD4^+^ T cells maintained their steady levels even in the absence of IL-2 (Fig. 1C). These early events also may correlate with the level of mitochondrial polarization [43], as successful T cell activation was associated with hyper-polarization of mitochondria, whereas initiation of T cell apoptosis was associated with mitochondrial depolarization [44]. Lack of IL-2 signaling in activated CD8^+^ T cells resulted in dramatic depolarization of mitochondrial membrane potential, whereas CD4^+^ T cells consistently maintained a hyper-polarized state of mitochondria under the same conditions (Fig. 1D). These results suggested that deficiency of IL-2 during the early phase of activation affected the survival of CD8^+^ T cells much more than CD4^+^ T cells. These results directly correlated in CD8^+^ T cells with highly reduced Bcl-2 expression, as well as membrane depolarization and thus, enhanced apoptosis.

### Neutralization of IL-2 during T-cell Priming Induces Apoptosis of CD8^+^ T Cells

**Figure 2:**
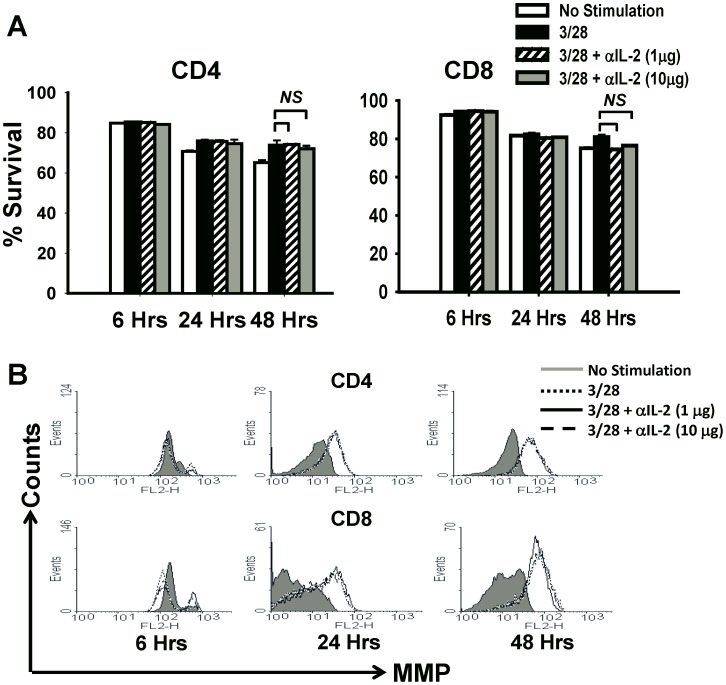
Constitutive expression of Bcl-2 rescues the survival of CD8^+^ T cells under IL-2 deprivation. CD4^+^CD25**^−^** and CD8^+^CD25**^−^** T cells were purified from Bcl-2 Tg mice and stimulated with anti-CD3/CD28 mAbs in the presence or absence of neutralizing IL-2 mAb at two different concentrations as indicated. Some cells were left unstimulated (no anti-CD3/CD28) and were used as negative controls. A total of 5×10^4^ cells per well were cultured in flat-bottom 96-well plates and harvested for analysis at the indicated time points. (**A**) Harvested cells were stained with Propidium Iodide (PI) to measure cell survival by flow cytometry. Bar graphs show the percentage survival in each group (n = 3) measured as the frequency of PI negative cells. (**B**) Histograms represent the mitochondrial membrane potential in the harvested cells measured by flow cytometry (with higher fluorescence depicting a hyper-polarized state of mitochondria) at indicated time points. The figure shows results from one representative experiment which was repeated three times with similar results. Bars indicate average value ± SD obtained from three culture wells (technical triplicates) in each experimental group.

To investigate whether the effects of IL-2 neutralization were due to apoptosis of activated T cells, we performed IL-2 blocking experiments with Bcl2-Tg mice, in which conventional T-cells are resistant to apoptosis [45]. We found that unlike wild-type B6 mice, CD8^+^ T cells from Bcl2-Tg mice do not show reduction in survival upon neutralization of IL-2 while CD4^+^ T cells from Bcl2-Tg mice, similar to B6 mice, maintain high rates of survival upon blockade of IL-2 signals (Fig. 2A). Overall, no significant difference in the rates of CD4^+^ or CD8^+^ T cell survival was observed at any of the measured time points under either concentration of neutralizing IL-2 mAb (Fig. 2A). These results also correlated with a state of hyper-polarized mitochondria in both CD4^+^ and CD8^+^ T cells, when activated under IL-2 neutralizing conditions (Fig. 2B). Since constitutive expression of the anti-apoptotic protein Bcl-2 rescued the survival of CD8^+^ T cells under IL-2 deprivation, our results suggest that IL-2 blockade causes apoptosis of activated CD8^+^ T cells rather than direct toxicity in B6 mice.

### Co-culture of CD8^+^ T Cells with CD4^+^ T Cells Rescues their Survival under IL-2 Deprivation

**Figure 3:**
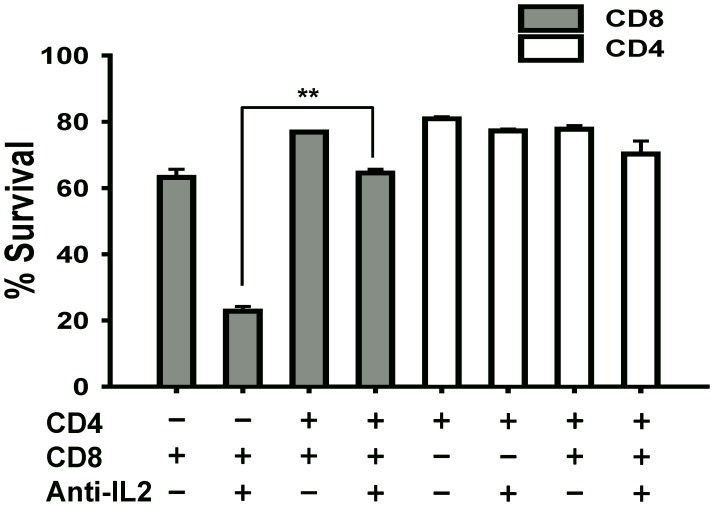
CD4^+^ T cells rescue the survival of CD8^+^ T cells upon co-culture in a trans-well. CD4^+^CD25**^−^** (1×10^5^) and CD8^+^CD25**^−^** (1×10^5^) T cells were isolated from wild-type B6 mice, and were cultured in the upper and lower chamber of a 24-well transwell plate respectively. Both populations of cells in upper and lower chambers were activated with 1∶1 (cells:beads) ratio of Dynabeads (Mouse T-activator CD3/CD28). In some wells, the two populations were cultured alone with Dynabeads in the lower chamber as indicated in the figure. Some culture wells were supplemented with 10 µg/mL anti-IL-2 mAb as shown in the figure. At 48 hrs post-culture, cells were harvested and stained with PI to analyze percent survival by flow cytometry. The bars (n = 3) show the frequency of PI negative cells within the CD4^+^ **(white bars)** and CD8^+^ **(grey bars)** T cells. The figure shows results from one representative experiment which was repeated three times with similar results. Bars indicate average value ± SD obtained from three culture wells (technical triplicates) in each experimental group (**denotes *p*<0.005).

Since CD4^+^ T cells were resistant to the effects of IL-2 deprivation (Fig. 1), we postulated that CD4^+^ T cells may produce IL-2 independent cytokines/growth factors during activation which may promote their survival under IL-2 blockade. To test this possibility, we cultured both CD4^+^ and CD8^+^ T cells in a trans-well plate separated by a semi-permeable membrane to allow sharing of soluble factors but preventing cell-cell contact between the two populations. Under these IL-2 deprived conditions (10 µg/mL anti-IL-2 mAb), the survival of CD8^+^ T cells was significantly rescued when CD4^+^ T cells were present in the same culture well compared to CD8^+^ T cells cultured alone (Fig. 3; 2^nd^ vs. 4^th^ gray bar). Consistent with our previous results, CD4^+^ T cells maintained high rates of survival with or without IL-2 neutralization as well as with or without CD8^+^ T cells in the same culture well (Fig. 3; white bars). The results from this experiment suggest that CD4^+^ T cells under IL-2 inhibition produced a soluble factor that not only promoted their own survival but also helped CD8^+^ T cells survival in the absence of IL-2 signals.

### IL-21 is the Only T Cell Produced Cytokine that Promotes CD8^+^ T Cell Survival under IL-2 Blockade

**Figure 4:**
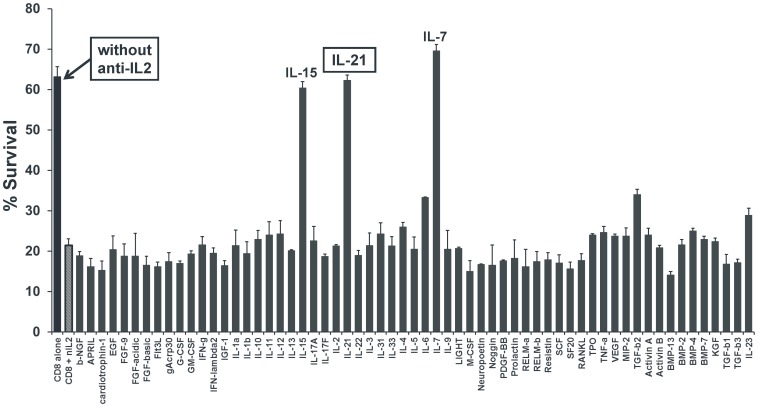
IL-21 rescues the survival of CD8^+^ T cells under IL-2 deprivation. CD8^+^CD25**^−^** T cells (5×10^4^) were purified from wild-type B6 mice and stimulated with anti-CD3/CD28 mAbs in the presence (**all grey bars**) or absence (**black bar**) of 10 µg/mL neutralizing IL-2 mAb. The cultures with anti-IL-2 were further supplemented with either 10 IU/mL (IL-2) or 10 ng/mL (all other cytokines), each of 61 different cytokines (as marked in the figure). The cells were harvested at 48 hrs and subsequently stained for PI for measuring cell survival. The bars graph shows frequency of PI negative cells under each culture condition as marked. The figure shows results from one representative experiment which was repeated three times with similar results. Bars indicate average value ± SD obtained from three culture wells (technical triplicates) in each experimental group.

To investigate which soluble factor/s can promote CD8^+^ T cell survival under IL-2 deprivation, we cultured naïve CD8^+^ T cells with or without neutralizing IL-2 mAb and then supplemented the cultures containing anti-IL2 with each of 61 different cytokines at concentrations indicated in the materials and methods. At 48 hours post-culture we measured cell survival and observed again that CD8^+^ T cell survival was significantly reduced under IL-2 blockade (Fig. 4; 1^st^ vs. 2^nd^ bar). However, supplementing the cultures with IL-15, IL-7 or IL-21 significantly rescued the survival of CD8^+^ T cells to levels similar to without IL-2 neutralization (Fig. 4). Among these, IL-21 is the only cytokine reported to be produced by CD4^+^ T cells. These results collectively suggest that CD4^+^ T cells under IL-2 blockade may switch to IL-21 production which may be involved in promoting their own survival as well as providing help to CD8^+^ T cells.

### IL-21 is Critical for Maintaining CD4^+^ and CD8^+^ T Cell Survival under IL-2 Inhibition

**Figure 5:**
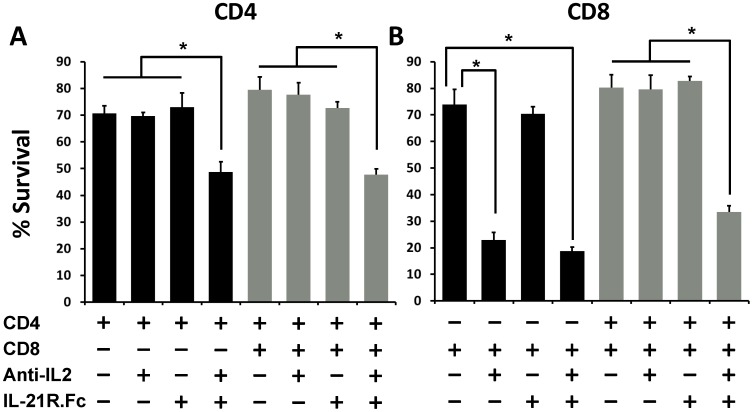
Neutralizing IL-2 and IL-21 simultaneously reduces the survival of both CD4^+^ and CD8^+^ T cells. CD4^+^CD25**^−^** and CD8^+^CD25**^−^** T cells were isolated from wild-type B6 mice, and were cultured together (2.5×10^4^ cells/well from each population) or individually (5×10^4^ cells/well) as indicated in a 96-well plate. The cells were stimulated with anti-CD3/CD28 mAbs in the presence or absence of anti-IL-2 and IL-21R.Fc as indicated. The cells were harvested at 48 hrs post-culture and subsequently stained for CD4, CD8 and PI for measuring cell survival. (**A**) The bars graph shows frequency of PI negative cells within the CD4^+^ T cell population when cultured alone (**black bars**) or with CD8^+^ T cells (**gray bars**). (**B**) Bars graph shows frequency of PI negative cells within CD8^+^ T cell population when cultured alone (**black bars**) or with CD4^+^ T cells (**gray bars**). The figure shows results from one representative experiment which was repeated three times with similar results. Bars indicate average value ± SD obtained from three culture wells (technical triplicates) in each experimental group (*denotes *p*<0.05).

To investigate whether IL-21 is a critical growth factor supporting CD4^+^ and CD8^+^ T cell survival under IL-2 blockade, we performed cultures of naïve CD4^+^ and CD8^+^ T cells either alone or together and with or without neutralizing IL-2 and IL-21. For neutralizing IL-21 in our cultures, we used a murine IL-21 receptor fusion protein (IL-21R.Fc) (generated by Pfizer Inc.). While neither neutralizing IL-2 nor neutralizing IL-21 alone affected CD4^+^ T cell survival, a combination of both neutralizing agents together reduced CD4^+^ T cell survival from 70–80% to 45–50% (Fig. 5A). Similar reduction in survival was observed when CD4^+^ T cells were cultured either alone or with CD8^+^ T cells (Fig. 5A). These results indicate that CD4^+^ T cells activated under IL-2 inhibiting conditions became dependent on IL-21 signaling for their survival. On the other hand, in cultures of CD8^+^ T cells alone, anti-IL2 was sufficient to reduce their survival while IL-21R.Fc did not have a significant effect on CD8^+^ T cell survival (Fig. 5B; black bars). However, when cultured along with CD4^+^ T cells, the CD8^+^ T cell survival was reduced only under conditions when both IL-2 and IL-21 signals were neutralized (Fig. 5B; gray bars). Thus, the protection from apoptosis conferred upon CD8^+^ T cells cultured under IL-2 deprivation conditions, by co-culture of CD4^+^ T cells was lost when IL-21 was also neutralized.

**Figure 6:**
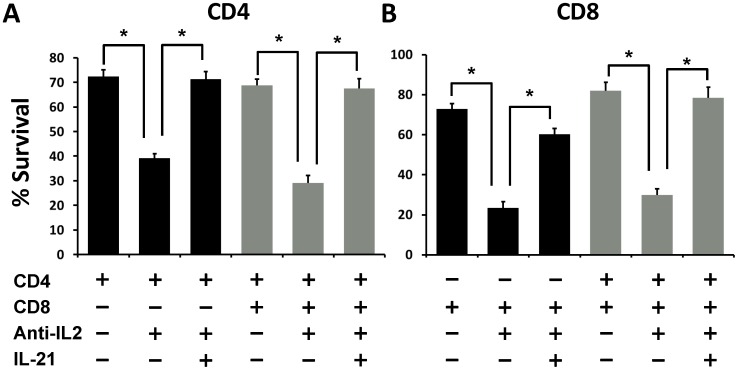
IL-21 is critical for maintaining survival of CD4 and CD8 T cells under IL-2 inhibition. CD4^+^CD25**^−^** and CD8^+^CD25**^−^** T cells were isolated from IL-21 KO mice, and were cultured together (2.5×10^4^ cells/well from each population) or individually (5×10^4^/well) as indicated in a 96-well plate. The cells were stimulated with anti-CD3/CD28 mAb’s in presence or absence of anti-IL-2 and recombinant murine IL-21 as indicated. The cells were harvested at 48 hrs post-culture and subsequently stained for CD4, CD8 and PI for measuring cell survival. (**A**) The bar graph shows frequency of PI negative cells within CD4^+^ T cell population when cultured alone (**black bars**) or with CD8^+^ T cells (**gray bars**). (**B**) Bars graph shows frequency of PI negative cells within CD8^+^ T cell population when cultured alone (**black bars**) or with CD4^+^ T cells (**gray bars**). The figure shows results from one representative experiment which was repeated three times with similar results. Bars indicate average value ± SD obtained from three culture wells (technical triplicates) in each experimental group (*denotes *p*<0.05).

To further validate the role of IL-21 in our studies, we cultured naïve CD4^+^ and CD8^+^ T cells from IL-21 KO mice with or without neutralizing IL-2 mAb. Interestingly, individual and co-cultures of both CD4^+^ and CD8^+^ T cells from IL-21 KO mice showed significantly reduced survival upon blockade of IL-2 signals (Fig. 6A & B). More importantly, addition of 10 ng/mL recombinant mouse IL-21 to these cultures rescued the survival of both CD4^+^ and CD8^+^ T cells under IL-2 deprivation (Fig. 6A & B). Thus, our results demonstrate a critical role for IL-21 signaling in promoting activated CD4^+^ and CD8^+^ T cell survival during conditions of IL-2 deprivation.

## Discussion

Current immunosuppressive therapies of T cell mediated ailments such as allograft rejection and some autoimmune diseases rely on small molecules that non-specifically inhibit T cell activation, but also block other non-immune cells. In contrast, selective inhibition of IL-2 signaling limits their effects to immune cells with a potential for exclusively blocking only the immune responses directed against alloantigens and autoantigens [20]; [21]. However, the real impact of IL-2 inhibition on T cells needs better understanding as IL-2-independent signaling pathways may regulate multiple aspects of T cell responses to auto- and allo- antigens. For instance, in our recent study we have demonstrated that activation of naïve CD4^+^ T cells under neutralizing IL-2 conditions leads to generation of iTreg precursors, which subsequently form fully functional and stable FoxP3 expressing Treg cells upon exposure to IL-2 [27]. Moreover, other groups have shown that primary and recall T cell responses to skin allografts and to vaccinia virus infection were intact in an IL-2Rβ−/− mouse lacking autoimmune diseases [32] suggesting that T cell expansion can occur in the absence of IL-2 by alternate mechanisms which are not yet defined. Herein, we present results demonstrating that IL-2 signaling was dispensable for the survival of CD4^+^ T cells (Figs. 1 & 2) as well as CD8^+^ T cells when they were co-cultured with CD4^+^ T cells (Fig. 3). Further, we have shown that under IL-2 deprivation conditions, IL-21 may act as the major growth factor promoting the survival of CD4^+^ and CD8^+^ T cells (Figs. 4, 5 & 6).

Although, administration of anti-IL2 mAb (clone S4-B6) in a complex with IL-2 in vivo has shown to have immune enhancing effects [46], such effects have not been reported in vitro, where the antibody is extensively used for neutralizing IL-2 applications [27], [41], [42]. This discrepancy in the effects of anti-IL2 mAb administration in vitro vs. in vivo is well recognized [46]. However, the exact mechanisms by which anti-IL2 mAb exerts its immune enhancing effects selectively in vivo remain unclear.

Low levels of IL-2 signaling may exist physiologically in various conditions. One such example is the human T1D patient and the non-obese diabetic (NOD) mouse. Both NOD mice and human T1D patients have polymorphisms in the *il2, il2ra* and *il2rb* genes which have been correlated with lower production of IL-2 and lower levels of IL-2 signaling [47]–[49]. For the most part, loss of IL-2 signaling in these hosts has been attributed to loss of Treg cell development, maintenance and suppressive function leading to lethal autoimmunity [28], [50]. However, what accounts for the substantial IL-2-independent expansion of self-reactive lymphocytes in these autoimmune hosts is not well understood. Our study provides insights into mechanisms by which CD4^+^ and CD8^+^ T cells may maintain effector function in the absence of IL-2 signaling. Since IL-21 was the only T cell produced cytokine (among the 61 tested cytokines) that improved the survival of activated CD8^+^ T cells under IL-2 neutralization (Fig. 4), it might be one of the key factors regulating autoimmune responses in these T1D hosts. Indeed, NOD mice deficient in IL-21 or IL-21R do not develop T1D unlike wild-type NOD mice among which 60–80% of the females develop T1D between 12–25 weeks of age [38], [39]. Thus, low dose IL-2 based therapies targeting expansion of Treg cells in type-1 diabetic hosts [51] may be supplemented with therapeutic agents to block IL-21 signaling to effectively prevent immune responses from pathogenic self-reactive T cells.

More recently, IL-21 signaling has gathered further attention in studies with chronic viral infections including the Human Immunodeficiency Virus (HIV) [52]–[54]. These studies emphasize the role of IL-21 in promoting CD4^+^ T cell help to effector CD8^+^ T cells and to antibody producing B cells during persistent viral infections when levels of IL-2 are low. In particular, impairment of the T follicular (T_FH_) cell help to B cells through IL-21 production was associated with HIV pathogenesis and reduced cellular proliferation [53]. Thus, it is possible that IL-21 is a key regulator of adaptive immune responses against foreign and self-antigens in scenarios where IL-2 signaling is not available to antigen activated cells. Such scenarios of immune cell activation in the absence of IL-2 signals may also exist in recipients of organ transplants, as most transplant recipients are treated with immunosuppressive drugs that work by lowering IL-2 production by T cells. It would be interesting to study the role of IL-21 in promoting rejection of allografts in spite of effective immunosuppression. Two groups recently tested neutralizing IL-21 in islet allograft models and have shown that IL-21 is a critical mediator of the late phase alloimmune response [55], [56]. Moreover, our results show that survival of CD4^+^ and CD8^+^ T cells cultured together was affected only when both IL-2 and IL-21 were blocked simultaneously (Figs. 5 & 6). These results may suggest a role for IL-21 in the late phase of alloimmune responses where sufficient IL-2 may not be available for activated T cells during this time.

In summary, we have shown that regulation of Bcl-2 dependent survival through the IL-2 signaling pathway seems different in CD4^+^ and CD8^+^ T cells when the two populations were cultured individually *in vitro*. However, co-culture of CD4^+^ and CD8^+^ T cells revealed that IL-2 signaling may be dispensable for the survival of activated T cells. Under these conditions the survival is dependent on alternate cytokines produced by CD4^+^ T cells during IL-2 neutralization. IL-21 emerged as the only T cell produced cytokine that promoted CD4^+^ and CD8^+^ T cell survival during conditions of IL-2 inhibition. Moreover, blocking IL-21 signals through IL-21R.Fc or by knocking out IL-21 along with IL-2 neutralization, lead to significant reduction in the survival of both CD4^+^ and CD8^+^ T cells, suggesting a critical role for IL-21 in regulating T cell responses during conditions of low IL-2 signaling. These findings expand our understanding of cytokine signaling in T cells as well as provide useful insights into designing better immune therapies for T cell mediated diseases by targeting cytokine signals.
